# Equity of the Essential Public Health Service in Rural China: Evidence from a Nationwide Survey of Hypertensive Patients

**DOI:** 10.12669/pjms.294.3773

**Published:** 2013

**Authors:** Donghua Zhou, Zhanchun Feng, Shasha He, Xi Sun, Caihui Ma, Benyan Lv, Xiong Zou

**Affiliations:** 1 Donghua Zhou, School of Medicine and Health Management, Tongji Medical College, Huazhong University of Science and Technology, Wuhan, China.; 2 Zhanchun Feng, School of Medicine and Health Management, Tongji Medical College, Huazhong University of Science and Technology, Wuhan, China.; 3 Shasha He, School of Medicine and Health Management, Tongji Medical College, Huazhong University of Science and Technology, Wuhan, China.; 4 Xi Sun, School of Medicine and Health Management, Tongji Medical College, Huazhong University of Science and Technology, Wuhan, China.; 5 Caihui Ma, School of Medicine and Health Management, Tongji Medical College, Huazhong University of Science and Technology, Wuhan, China.; 6 Benyan Lv, School of Medicine and Health Management, Tongji Medical College, Huazhong University of Science and Technology, Wuhan, China.; 7 Xiong Zou, Maternal and Child Health Hospital of Guangxi Zhuang Autonomous Region, Nanning, Guangxi, PR China.

**Keywords:** Essential public health services, Equity, Hypertensive patients, Rural China

## Abstract

***Objectives:*** To explore healthcare disparities in rural China two years after the implementation of the Essential Public Health Service (EPHS) reform in 2009.

***Methods:*** A cross-sectional study was conducted by surveying 930 hypertension patients (HPs) from different regions in rural China in 2011. The percentages of patients using recommended four or more follow-up visits in a year were calculated by patient socio-demographic characteristics and statistically examined using chi-square and logistic regression to uncover disparities and correlated factors in EPHS use.

***Results:*** The rates were not significantly different by age, gender, education, insurance status or income, but significantly different by region and hypertension history (p<0.01). Higher rates were also observed on patients who sought actively follow-up service at clinics, making appointment for the next follow-up with doctors, awareness of the need of follow-up, more satisfied with the follow-up services, and better medication adherence (p<0.01).

***Conclusions:*** There were no disparities observed among HPs in the use of follow-up services, suggesting that the reform has to some extent achieved its goal in ensuring equal access to EPHS. In this regard, regional implementation of the national policies and improvement of EPHS management at local level should be further improved.

## INTRODUCTION

Sweeping economic reforms in China over the past three decades have brought about unprecedented economic prosperity. However, they have also significantly widened the income and social inequality among Chinese people. The rural-urban divide in health and healthcare is especially evident in the fact that the average life expectancy of urban residents is 73 years, 8 years longer than that of rural residents.^1^ The disparities across rural areas are equally serious, where infectious diseases, endemic diseases and malnutrition are still prevalent, chronic conditions are on the rise, with many families in poverty or falling into poverty due to illness. Uneven developments in economics and health systems make access to essential health services a greater challenge in some areas and for some families than others.^2^^-^^4^ Many studies have shown that age, gender, educational background and income significantly correlated with rural residents’ health and healthcare^5^^,^^6^, with poverty being the strongest predictor for more severe health problems and lower healthcare utilization.^7^^-^^12^

Such disparities are causing widespread discontent and increasingly threatening social stability. Therefore, this issue has become a grave concern for the Chinese government. The government has in recent years initiated a series of reforms targeted at removing these disparities. The most important step taken was the release of the “Deepening Healthcare and Pharmaceutical Systems Reform Plan” in April 2009, which set a goal to achieve universal health coverage for all urban and rural residents by 2020 and to provide safe, effective, affordable and equitable health services for all. The plan called for immediate actions to: 1) construct a basic healthcare financing system such as the New Rural Medical Cooperative System (NRMCS); 2) establish a more efficient national pharmaceutical dispensing system; 3) improve the existing basic medical and health services infrastructure; 4) promote equal access to essential public health services (EPHS);^13^^,^^14^ and 5) strengthen public hospitals’ reform. Since 2009, the government has invested an estimated 850 billion Chinese Yuan towards achieving these objectives.^15^

Ensuring universal access to EPHS is central to these reforms and consistent with the global initiatives by WHO. In addition to the resources invested in general infrastructure and other aspects of healthcare system development, the government has made a commitment to provide free EPHS to the entire Chinese population since July 2009, covering: 1) health records management; 2) health education; 3) comprehensive health care for children aged 0-36 months; 4) comprehensive prenatal care; 5) comprehensive care for the elderly; 6) vaccination; 7) report and action on infectious disease; 8) management of hypertensive patients (HPs); 9) management of type 2 diabetes mellitus patients; and 10) comprehensive care for severe mental ill patients. All the EPHS items in rural areas are provided mainly by village clinics and township hospitals, and national guidelines and performance standards for EPHS are established and regularly evaluated.^16^

Although the rationale for these reforms and the expectations are clear,^17^^,^^18^ little research illustrates their actual impact. This study is the first attempt to assess to what extent these reforms have achieved their goals in eliminating disparities in the use of EPHS, based on a 2011 nationwide survey of HPs on their use of recommended follow-up services. The subjects were chosen for the following three reasons: 1) More than 200 million Chinese are hypertensive;^19^ 2) the importance and guidelines for regular follow-up visits to monitor blood pressure, ensure medication adherence and educate patients about diets and lifestyles to avoid exacerbation and complications were well established; and 3) management of HPs explicitly listed the government’s July 2009 EPHS plan. Therefore, the study based on HPs could provide generalizable evidence on the overall success of China’s recent reforms in reducing disparities in EPHS.

## METHODS

**Fig.1:**
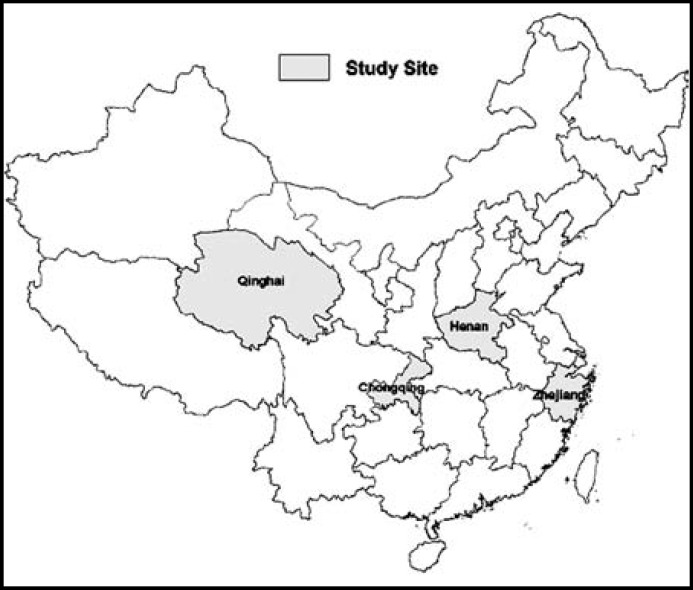
Distribution map of the study sites

***Study population: ***A multistage stratified random sampling method was used to survey HPs aged 35 or above across China. First, all provinces were grouped into four regions according to the Human Development Index, and one province was randomly chosen from each region. The selected provinces were Zhejiang (Eastern China), Henan (Central China), Chongqing (Western China) and Qinghai (Northwest China) province, which each represent four different economy development levels in China. Based on the stratified multi-stage sampling method, a total of 108 villages from 36 townships of 12 counties of four provinces were selected following the sequence of provinces-counties-townships-villages area. In each sampled village, the respondent-driven sampling method was used to find participants: the first participant was recommended by the village doctor; then, the first participant recommended the next participant after the interview. This procedure was followed repeatedly until the tenth or the last HP in the village was interviewed. (Fig.1)

***Questionnaire:*** The survey questionnaire consisted of two parts: socio-demographic information and utilization of follow-up services. The socio-demographic information requested gender, age, educational level, family income in 2010-2011, medical insurance, resident regions, distance to closest healthcare facilities, and history of hypertension. Information on service utilization included the number of times of seeing doctors for hypertension in the previous year, content of the services, follow-up service mode, whether doctor made appointment for the next follow-up, patients’ satisfaction over the services, awareness of the need for follow-up, and adherence to taking medicine regularly.

Participants (N=930) were interviewed face-to-face by students from the Tongji Medical College and staff of local healthcare institutions from July to December in 2011. This study was approved by the Ethics Committee of Tongji Medical College. Informed consent was obtained from all participants.

***Data Analysis:*** According to the National EPHS Standards published by the Ministry of Health (2009), public health service providers should follow-up HPs at least four times per year.^16^ Accordingly, the rates of HPs who received four or more follow-up services were calculated within a year and, as dependent variable, compared across patient socio-demographic and other independent variables. Logistic regressions were conducted to ascertain EPHS disparities independently associated with income variable. Upon discovering substantial regional differences in follow-up services and recognizing that region also represented income differences among HPs, this study ran two logistic regressions in parallel, one with region as control variable and one without region, to gain insight into the income effects on EPHS by the indicator of region and by reported family income. The associations between follow-up service use and service contextual variables were analyzed to further explore the differences in EPHS among HPs. All data were analyzed using SPSS 18.0 (Chicago, IL, USA).

## RESULTS

***Socio-demographic characteristics:*** Table-I presents the demographic characteristics of the participants, who were mostly aged 65 or above, with education below middle school, with coverage by NRCMS (The universal coverage rate was more than 90%). Most participants had been diagnosed with hypertension for more than one year. The number of male participants was slightly less than that of female participants.

***Use of recommended follow-up services by patient characteristics: ***The overall rates of receiving four or more follow up services a year was 71.1%. Table-I also shows the rates of receiving four or more follow-up visits vary very little by patient socio-demographic factors, which is supported by the result that some of the socio-demographic factors including age, gender, educational level, income and medical insurance, did not significantly influence HPs utilizing EPHS equally (p>0.05). Regional differences are striking, however. The northwestern province’s rate is 35% higher than that of the eastern province. In addition, patients living closer to healthcare facilities appeared to have significantly higher rates of follow-up visits.

Table-II displays the result of logistic regressions. Controlling for other factors, HPs in the northwestern province are nearly five times more likely to receive adequate follow-up services than those in the eastern province. The other significant variable is hypertension history; patients suffering from hypertension for more than five years were twice likely to receive adequate follow-up visits than patients whose hypertension history was less than one year. All other socio-demographic variables were insignificant in the regression, and the results changed little when region was removed from the regression analysis.

***Correlation factors with improving follow-up use among HPs:*** Table-III shows that some other care process-related factors influence HPs' follow-up services use. Patients visiting clinics actively had a higher rate of getting adequate follow-up service than those passively waiting for doctors’ telephone calls or house visits. Making appointment for next visit, patient awareness of the importance of regular follow-up, higher satisfaction with follow-up services (technology service quality, interpersonal quality and environmental quality) and medication adherence were also associated with increased follow-up service use.

**Table-I:** The use of hypertension follow-up services by patient characteristics

			*Follow-up visits in the past year*		
*Predictor*	*N*	*%*	*% ≥4 visits a year* *(N=662)*	*χ2*	*p-value*
*Age groups (years) *					
35-50	97	10.4	69.1		
50-65	301	32.4	72.1		
65+	532	57.2	71.1	.337	.845
*Gender *					
Male	413	44.4	70.5		
Female	517	55.6	71.8	.189	.664
*Level of education*					
No formal school	447	48.1	70.2		
Elementary	296	31.8	72.0		
Middle school	122	13.1	70.5		
High school and above	65	7.0	75.4	.866	.834
*2010-2011 income *					
<5,000 RMB	181	19.5	74.0		
5,000-10,000 RMB	147	15.8	72.8		
10,000-15,000 RMB	137	14.7	69.9		
15,000-20,000 RMB	103	11.1	75.9		
>20,000 RMB	362	38.9	68.0	4.453	.348
*Medical Insurance*					
NRCMS	841	90.4	71.5		
Others	89	9.6	68.5	.335	.563
*Regions of residence*					
Zhejiang - Eastern China	210	22.6	50.5		
Henan - Central China	138	14.8	83.3		
Chongqing - Western China	237	25.5	62.0		
Qinghai - Northwest China	345	37.1	85.2	96.643	**.000**
*Distance to closest healthcare facilities*					
<1 km	736	79.1	73.9		
>1 km	194	20.9	60.8	12.821	**.000**
*History of hypertension*					
<1 year	70	7.5	61.4		
1-5 year	433	46.6	74.1		
> 5 year	427	45.9	69.8	5.489	.064

Refers to non-New Rural Cooperative Medical System （NRCMS） such as private medical insurance or no medical insurance and so on.

**Table-II:** Logistic regression on having had 4 or more follow-up visits in the past year

*Explanatory Variable*	*Regression without region*		*Regression with region*	
	*Odds Ratio*	*95% CI*	*Odds Ratio*	*95% CI*
*Age groups (years)*				
* 35-50*	1		1	
50-65	1.508	（.838, 2.714）	1.613	(.881, 2.952)
65+	1.500	（.862, 2.612）	1.555	(.881, 2.743)
*Gender *				
Male	1		1	
Female	1.171	（.826, 1.661）	1.227	(.856, 1.756)
*Level of education*				
No formal school	1		1	
Elementary	1.328	（.896, 1.969）	1.409	(.938, 2.115)
Middle school	1.294	（.753, 2.224）	1.326	(.761, 2.309)
High school and above	1.671	（.766, 3.646）	1.629	(.732, 3.625)
*Medical Insurance*				
NCMS	1		1	
Others	1.616	(.773, 3.377)	1.700	(.815, 3.547)
*2010-2011 income*				
<5,000 RMB	1		1	
5,000-10,000 RMB	.641	(.362, 1.136)	.712	(.399, 1.272)
10,000-15,000 RMB	1.151	(.632, 2.097)	1.433	(.769, 2.670)
15,000-20,000 RMB	.741	(.399, 1.376)	.821	(.434, 1.554)
>20,000 RMB	.807	(.502, 1.298)	.897	(.547, 1.471)
*Regions of residence*				
Zhejiang - Eastern China			1	
Henan - Central China			2.117	(1.051, 4.265)*
Chongqing - Western China			1.739	(1.077, 2.809)*
Qinghai - Eastern Northwest China			4.760	(2.784, 8.139)**
*Distance to closest healthcare facilities*				
<1 km	1		1	
>1 km	.718	(.480, 1.074)**	.854	(565, 1.292)
*History of hypertension*				
<1 year	1		1	
1-5 year	2.119	(1.151, 3.900)*	2.430	(1.296, 4.555)**
> 5 year	1.773	(.962, 3.270)	2.185	(1.156, 4.129)*

* P<0.05;    ** p<0.01

**Table-III:** Correlates of HPs follow-up services

*Predictors*	*N*	*%*	*% ≥4 visits* *a year* *(N=662)*	*χ2*	*p-value*
*Primary mode of follow-up*					
Patient visits clinic	733	78.8	73.5		
Doctor telephone follow-up	31	3.3	58.1		
Doctor house visit	166	17.9	65.8	9.664	.008
*Doctor made appointment for next follow-up*					
Usually yes	483	51.9	85.9		
Usually no	447	48.1	63.3	106.415	.000
Patient awareness of the need of follow-up					
No need if there is no symptom	271	29.1	61.3		
Need, but no time	147	15.8	71.4		
Need regular follow-up	512	55.1	76.4	19.735	.000
*Patient satisfaction over the follow-up services*					
*Technology quality *					
Below average	517	55.6	62.3		
Above average	413	44.4	82.3	44.959	.000
*Interpersonal quality*					
Below average	466	50.1	61.2		
Above average	464	49.9	81.2	45.752	.000
*Environment quality *					
Below average	509	54.7	61.9		
Above average	421	45.	82.4	47.375	.000
*Medication adherence*					
Yes	733	78.8	73.9		
No	197	21.2	60.9	12.849	.000

## DISCUSSION

*Main findings and implications: *This study explored demographic factors (i.e., age and gender), social structure (i.e., educational level) and enabling factors (i.e., income and medical insurance) that are commonly used to assess disparities in health service utilization^20^ had no significant disparities in using recommended follow-up services among HPs with different levels of family income. This implies that regardless of the region the patients are located in, poor people and rich people received the same level of EPHS. Therefore, the analysis suggests that poor people have equitable access to EPHS utilization with rich people, which contrasts with many earlier studies that revealed significant disparities in health and health services utilization, especially income-related disparities.^5^^-^^12^ Results from this study suggest that the reforms initiated since 2009 may have, to some extent, achieved the goal of universal and equal access to EPHS in rural China. The establishment of the NRCMS in rural areas may have increased overall access to healthcare and increased the interaction among rural residents and the healthcare provider. The government’s July 2009 initiative that offered free EPHS may have further reduced economic obstacles for poorer families, resulting in increased equity in EPHS. The specific emphasis on patient records management, health education and comprehensive care for prevalent conditions may also have helped to increase awareness and use of EPHS.

One surprising finding is that the more developed, richer eastern region had substantially lower rates of adequate EPHS use than that the less developed, poor western region (OR=4.760; 95%CI=2.784, 8.139). There are several possible explanations. Chinese central government has provided more fiscal aid to healthcare workers in the relatively less developed central and western areas. It was reported that the proportion of government financial subsidies to the poor township hospitals in middle and west areas was 44.9% of their annual revenue,^21^ as a relatively larger portion of their income comes from the government and their patients are less able to pay for medical care. This might have stimulated township hospitals and village doctors in the western region to pay greater attention than their counterparts in the eastern region in providing EPHS. In contrast, because their patients have expendable income, township hospitals and village doctors in the eastern region may have focused more on providing medical care that is more profitable and been less motivated to follow the government’s call for increasing EPHS. The results indicate that different strategies are needed for different regions to ensure consistent implementation of the national reforms in ensuring universal EPHS.^22^

This study also reveals some contextual or process-related factors that are significantly associate poor use of EPHS. For example, the study suggests that educating patients about the importance of EPHS, about their active pursuit of the services and about medication adherence could significantly improve EPHS use. Furthermore, doctors at township hospitals and village clinics should be stimulated to change their service model to improve their service quality and to enhance their service capability. They should go to villagers’ homes to provide patients with follow-up service actively and not just wait in their offices for patients. Additionally, the health environment should also be improved. In a word, encouraging doctors to make an appointment for the next visit, to improve their services and to increase patients’ satisfaction could also lead to better utilization of EPHS.

***Limitations: ***This study relied on some self-reported answers, subjected to self-assessment bias and recall bias. Another limitation is that, albeit offering evidence on overall success, this study provides no insights into the effectiveness, cost-effectiveness and sustainability of these initiatives. As the reforms continue to deepen, more efforts should be made in more systematic evaluation, with better validated indicator metrics and more comprehensive study design.^23^^,^^24^

## CONCLUSION

This nationwide survey offers the first evidence that a series reforms in recent years may have to a great extent achieved equity in EPHS use in rural China. It also points to the direction for further efforts in both consistent regional implementations of the national policies and improvement of EPHS management at the local level.^25^

## Authors Contributions:

DHZ conducted the data analysis, drafted the manuscript and contributed to subsequent revisions. 

ZCF conceived the idea for the study, participated in study design, and contributed to the data analysis, drafting and revising of the manuscript. Other authors contributed to implementing the study, analyzing the data, and editing of the final manuscript. All authors read and approved the final manuscript.

## Notes

***Declaration of conflicting interests: ***The author(s) declared no potential conflicts of interest with respect to the research, authorship, and/or publication of this article.
